# Antibiotic Resistance via Bacterial Cell Shape-Shifting

**DOI:** 10.1128/mbio.00659-22

**Published:** 2022-05-26

**Authors:** Nikola Ojkic, Diana Serbanescu, Shiladitya Banerjee

**Affiliations:** a Department of Physics and Astronomy, Institute for the Physics of Living Systems, University College London, London, United Kingdom; b School of Biological and Behavioural Sciences, Queen Mary University of London, London, United Kingdom; c Department of Physics, Carnegie Mellon University, Pittsburgh, Pennsylvania, USA; University of Washington

**Keywords:** antibiotic resistance, growth physiology, mathematical modeling, bacterial morphogenesis, drug transport

## Abstract

Bacteria have evolved to develop multiple strategies for antibiotic resistance by effectively reducing intracellular antibiotic concentrations or antibiotic binding affinities, but the role of cell morphology in antibiotic resistance remains poorly understood. By analyzing cell morphological data for different bacterial species under antibiotic stress, we find that bacteria increase or decrease the cell surface-to-volume ratio depending on the antibiotic target. Using quantitative modeling, we show that by reducing the surface-to-volume ratio, bacteria can effectively reduce the intracellular antibiotic concentration by decreasing antibiotic influx. The model further predicts that bacteria can increase the surface-to-volume ratio to induce the dilution of membrane-targeting antibiotics, in agreement with experimental data. Using a whole-cell model for the regulation of cell shape and growth by antibiotics, we predict shape transformations that bacteria can utilize to increase their fitness in the presence of antibiotics. We conclude by discussing additional pathways for antibiotic resistance that may act in synergy with shape-induced resistance.

## OPINION/HYPOTHESIS

**FIG 1:**
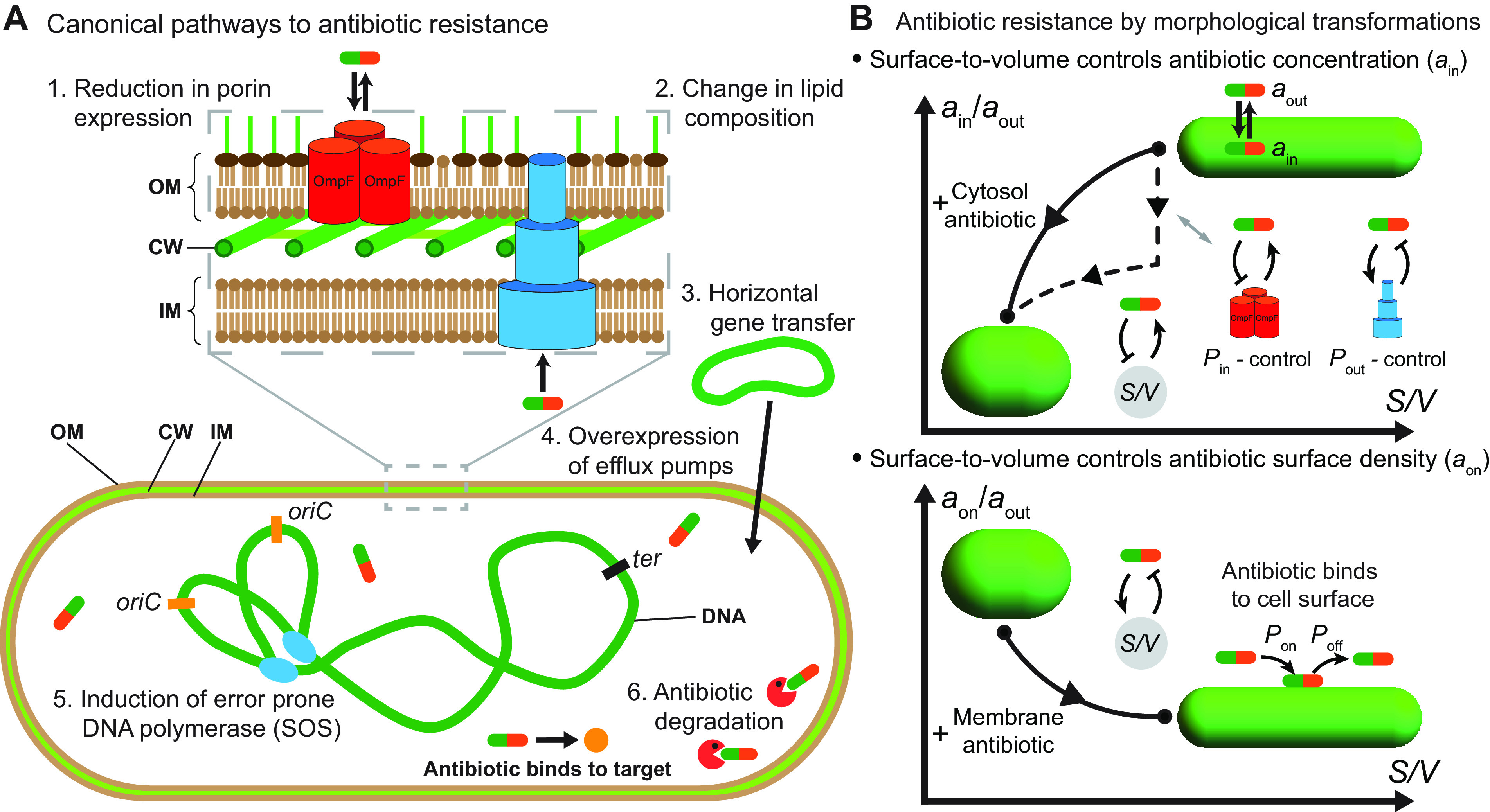
Mechanisms of antibiotic resistance at the single-cell level. (A) Canonical mechanisms of antibiotic resistance result in reduced intracellular antibiotic concentrations or reduced antibiotic binding affinities for their targets. Six pathways are shown. (1) Reduction in porin expression. The trimer of the passive transporter porin OmpF is shown in orange. (2) Lipid composition affects antibiotic translocation across the membrane. (3) Acquisition of resistance genes through horizontal gene transfer. (4) Overexpression of efflux pumps depletes intercellular antibiotics. The multidrug efflux pump AcrAB-TolC is shown in blue. (5) During the SOS response, bacteria express error-prone DNA polymerases that increase the random mutation rate. (6) Antibiotics are neutralized by specific proteins, shown as red Pac-Man symbols. Shown is an example of antibiotic binding to the target. OM, outer membrane; IM, inner membrane; CW, cell wall. (B) Mechanism of antibiotic resistance via cell shape transformation. (Top) Schematic showing a pathway (solid line) for reductions in the intracellular antibiotic concentration (*a*_in_) via changes in the cell surface-to-volume ratio (*S*/*V*) when the bacterial cell is exposed to a constant extracellular antibiotic concentration (*a*_out_). Pathways for reducing intracellular antibiotic concentrations by solely reducing *S*/*V*, lowering porin expression, or overexpressing efflux pumps are shown with dashed lines. In the schematic, *P*_in_ and *P*_out_ represent the membrane permeability coefficients in the inward and outward directions, respectively. (Bottom) For membrane-bound antibiotics, an increase in *S*/*V* decreases the antibiotic surface density (*a*_on_). Here, *P*_on_ and *P*_off_ are the rates of antibiotic binding to the membrane and unbinding from the membrane, respectively.

Antibiotic resistance is one of the major threats to human society. It has been estimated that each year, 700,000 people die as a consequence of infections caused by resistant bacteria, prompting an urgent response in order to prevent devastating global effects within generations (1). To understand the mechanisms of antibiotic resistance, we need to better understand how antibiotics physically penetrate bacterial cells, how antibiotics bind to their targets, what damage antibiotics cause to bacterial physiology, and, ultimately, how this damage leads to cell death (2, 3). To become antibiotic resistant, bacteria have developed multiple strategies. Resistance is commonly attained via reducing the intracellular concentration of the antibiotic or by reducing antibiotic binding affinities for their specific intracellular targets (Fig. 1A) (4). Various different pathways to antibiotic resistance have been described (5), including decreases in antibiotic influx by reductions in porin expression (6), modulation of the membrane lipid composition (7), induction of horizontal gene transfer (8), increases in antibiotic efflux by increasing efflux pump expression (9), the SOS response (10), and direct inactivation of antibiotics (11). However, the role of cell size, shape, and growth physiology in antibiotic resistance remains poorly understood.

Recent studies have shown that bacteria undergo a wide variety of cell morphological changes in response to antibiotics (12–18). These morphological changes (Fig. 1B) commonly occur via changes in the cell size, surface-to-volume ratio (*S*/*V*), or curvature (13, 15, 18). For instance, the Gram-negative bacteria Escherichia coli and Caulobacter crescentus and the Gram-positive bacterium Listeria monocytogenes decrease their *S*/*V* upon treatment with ribosome-inhibitory and cell wall-targeting antibiotics (15). It has also been shown that the Gram-negative human pathogen Pseudomonas aeruginosa makes a transition from rod-shaped cells to spherical cells upon treatment with β-lactams (14). However, it is not clear if these shape changes represent a passive physiological response to biochemical changes caused by the antibiotic or if these are active shape changes that promote bacterial fitness for surviving antibiotic exposure. While the roles of cell size and shape in bacterial growth and motility have been characterized (15, 17, 19–22), the effect of cell shape on antibiotic resistance remains poorly understood.

Changes in cell size and shape can be physiologically beneficial to antibiotic-treated bacteria in several ways. An increase in the cell volume can dilute the antibiotic concentration inside a cell, thereby promoting cell growth. A reduction in *S*/*V* can reduce the flux of antibiotics coming into the cell (Fig. 1B). An increase in *S*/*V* can also provide adaptive benefits by increasing the rate of nutrient uptake or by increasing the rate of antibiotic efflux. An outstanding question is how bacteria modulate their cell morphologies to promote resistance to different types of antibiotics that target different cellular components. In this article, we use quantitative modeling and data analysis to propose that cell shape-shifting via changes in *S*/*V* promotes antibiotic resistance by effectively diluting the intracellular antibiotic concentration. In particular, by developing a mathematical model for antibiotic transport and kinetics coupled to bacterial cell shape and growth, we show that by changing *S*/*V* of the cell, bacteria can effectively dilute the intracellular antibiotic concentration by decreasing antibiotic influx (Fig. 1B). The model allows us to predict the quantitative range for antibiotic dilution via reduction in *S*/*V*. The model also explains how bacteria can increase *S*/*V* to promote resistance to membrane-targeting antibiotics (Fig. 1B), in agreement with experimental data (13). To understand how antibiotics induce cell shape transformations, we develop a whole-cell model for bacterial growth and shape regulation using the particular example of ribosome-targeting antibiotics for which the biochemical pathways are quantitatively characterized. Using this model, we show that antibiotic-induced shape changes are dependent on nutrient availability such that it is beneficial for cells to increase *S*/*V* in nutrient-rich media (to promote nutrient influx) and to decrease *S*/*V* in nutrient-poor media (to inhibit antibiotic influx). We conclude by discussing additional mechanisms of countering antibiotics via regulating metabolic pathways and membrane porin and efflux pump expression that may act in synergy with shape-induced resistance.

## ANTIBIOTIC-INDUCED CELL SHAPE CHANGES IN ROD-SHAPED BACTERIA

**FIG 2:**
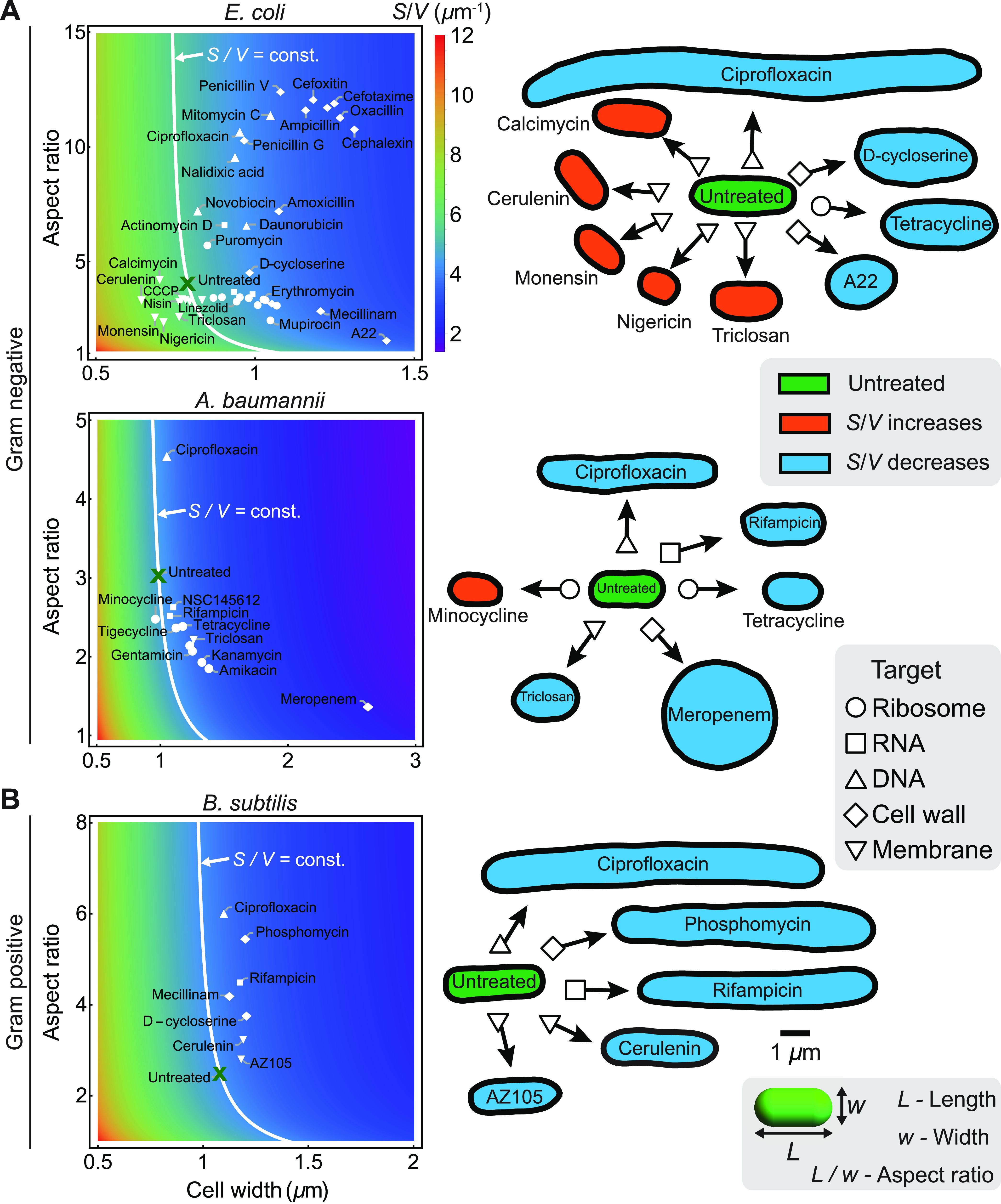
Changes in the cell shape and surface-to-volume ratio of rod-shaped bacteria under different antibiotic treatments. (Left) Heat map of the cell surface-to-volume ratio (*S*/*V*) as a function of the cell width and aspect ratio, overlaid with experimental data for the population-averaged cell shape under antibiotic treatments targeting different cellular components: ribosomes, RNA, DNA, the cell wall, and membranes. White lines represent a constant *S*/*V* corresponding to untreated cells. (Right) Typical cell contours for morphological responses to antibiotic treatments. *S*/*V* increase is shown in red, *S*/*V* decrease is in blue, and untreated cells are in green. (Data are taken from references 13, 55, and 56.) Cell contours were extracted using the ImageJ plug-in JFilament (57). (A) *S*/*V* for the Gram-negative bacteria E. coli and A. baumannii as a function of the cell width and cell aspect ratio. E. coli decreases *S*/*V* for all antibiotics apart from membrane-targeting ones, for which *S*/*V* increases. A. baumannii decreases *S*/*V* for all antibiotics apart from the ribosome-targeting antibiotic minocycline, for which *S*/*V* slightly increases. CCCP, carbonyl cyanide *m*-chlorophenylhydrazone. (B) *S*/*V* for the Gram-positive bacterium B. subtilis decreases for all antibiotics (22).

**FIG 3:**
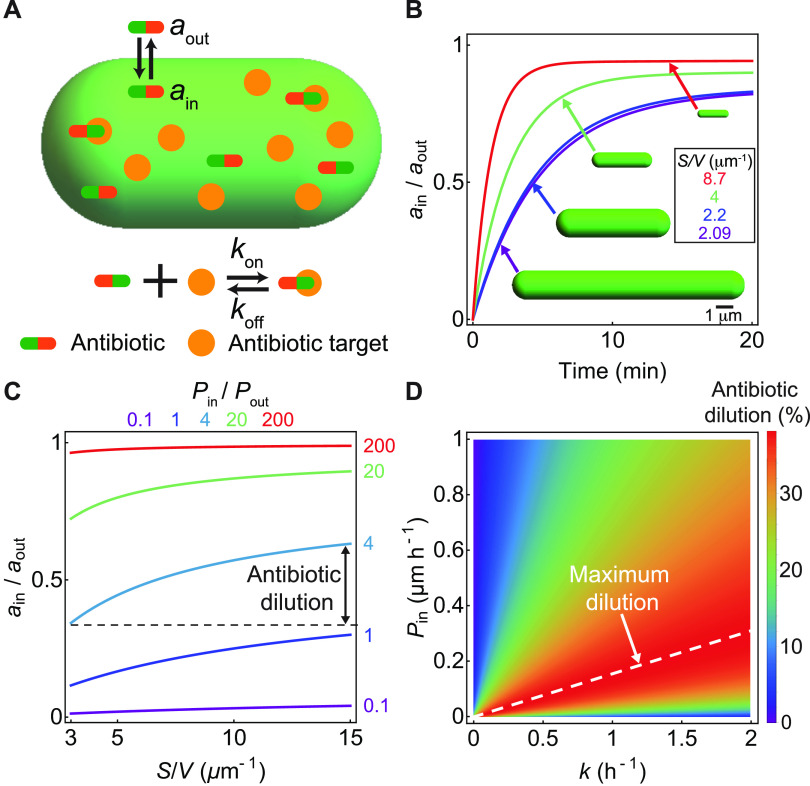
Cell shape-dependent dynamics of the antibiotic concentration in a bacterial cell. (A) Schematic of the model for antibiotic uptake and substrate binding/unbinding inside a bacterial cell. (B) Relative antibiotic concentration inside a cell versus time for different values of *S*/*V*, with all other parameters kept fixed (*P*_in_ = 5 μm h^−1^, *P*_out_ = 0.1 μm h^−1^, *k*_on_ = 1 μM^−1^ h^−1^, *k*_off_ = 0 h^−1^, *k_x_* = 1 μM h^−1^, and *k *= 1 h^−1^). (C) Steady-state antibiotic concentration normalized by *a*_out_ versus *S*/*V* for different values of *P*_in_/*P*_out_. For the cases *P*_in_ ≫ *P*_out_ or *P*_in_ ≪ *P*_out_, antibiotic dilution due to *S*/*V* changes is negligible. The range of *S*/*V* values is chosen to cover a range similar to that in Fig. 2. (D) Heat map of percent antibiotic dilution (δ) for different values of *k* and *P*_in_, setting *P*_out_ equal to 0, (*S*/*V*)_min_ equal to 3 μm^−1^, and (*S*/*V*)_max_ equal to 15 μm^−1^. The maximum antibiotic dilution is obtained when $P_{\text{in}}\sqrt{{\left(S/V\right)}_{\text{min}}{\left(S/V\right)}_{\text{max}}}=k$, as shown by the dashed line.

To understand the effect of antibiotics on bacterial cell shape, we first analyzed the morphological data for E. coli cells treated with 42 different antibiotics belonging to 5 different categories based on their binding targets (see Fig. 3B; see also Fig. S1 in the supplemental material) (13). While the cell volume and surface area increased for antibiotics that target DNA, RNA, ribosomes, or the cell wall, membrane-targeting antibiotics induced reductions in both the surface area and the volume of the cell (Fig. S1). Surprisingly, all antibiotics decrease *S*/*V* except for membrane- and membrane transport-targeting antibiotics, which increase *S*/*V* (Fig. 2A). Similarly, a decrease in *S*/*V* was previously observed in cells treated with cell wall-targeting antibiotics (A22, amdinocillin, and fosfomycin) (23), and an increase in *S*/*V* was previously observed for the membrane-targeting antibiotic cerulenin (24). We find that the Gram-negative bacterium Acinetobacter baumannii also decreases *S*/*V* for most antibiotics, including the membrane-targeting antibiotic triclosan (Fig. 2B). For the Gram-positive bacterium Bacillus subtilis, with a thick, less plastic cell envelope (25, 26), *S*/*V* decreases for all groups of antibiotics (Fig. 2C) (22). While these shape changes could represent a passive side effect of the antibiotic, they could also represent adaptive responses to counter the antibiotic’s action. In particular, we note that *S*/*V* is one of the key physical parameters that regulate nutrient influx and waste efflux (19) as well as the rates of influx and efflux of antibiotics. To quantitatively understand the role of *S*/*V* in regulating antibiotic flux across the cell membrane, we developed a mathematical model of antibiotic transport into a rod-shaped bacterial cell, with binding/unbinding interactions with its specific targets in the cytosol or on the membrane.

10.1128/mbio.00659-22.2 FIG S1 Surface area and volume of E. coli cells under treatment with different antibiotics. (A) Cell surface area represented with color as a function of the cell width and cell aspect ratio. Red represents an increase and blue represents a decrease in the surface area relative to untreated cells. The black dashed line represents a constant surface area as measured for untreated cells. (B) Cell volume represented with color as a function of the cell width and aspect ratio. The dashed black line represents a constant volume as measured for untreated cells. Download FIG S1, EPS file, 2.2 MB. Copyright © 2022 Ojkic et al. 2022 Ojkic et al. https://creativecommons.org/licenses/by/4.0/ This content is distributed under the terms of the Creative Commons Attribution 4.0 International license.

## THE CELL SURFACE-TO-VOLUME RATIO REGULATES INTRACELLULAR ANTIBIOTIC CONCENTRATIONS

When antibiotics are passively translocated into the cell thorough membrane porins or lipids (Fig. 3A), antibiotic transport is diffusion limited, and flux is given by Fick’s law (27, 28). The dynamics of the intracellular antibiotic concentration, *a*_in_, and the substrate concentration, *x*, are given by

(1) $$\frac{\text{d}a_{\text{in}}}{\text{d}t}=P_{\text{in}}(a_{\text{out}}-a_{\text{in}})\frac{S}{V}-P_{\text{out}}a_{\text{in}}\frac{S}{V}-k_{\text{on}}a_{\text{in}}x+k_{\text{off}}(x_{0}-x)-ka_{\text{in}}$$

(2) $$\frac{\text{d}x}{\text{d}t}=k_{x}+k_{\text{off}}(x_{0}-x)-k_{\text{on}}a_{\text{in}}x-kx\;$$

where *a*_out_ is the concentration of the antibiotic in the extracellular medium, *x*_0_ is the substrate concentration in the absence of the antibiotic, *k_x_* is the rate of substrate production, *k*_on_ is the rate of binding of the antibiotic to the substrate, *k*_off_ is the antibiotic unbinding rate, and *P*_in_ and *P*_out_ are the membrane permeability coefficients in the inward and outward directions, respectively. The last term on the right-hand side of equations 1 and 2 represents the dilution of the antibiotic and the substrate due to cell growth, where *k* is the bacterial growth rate. Using model parameters provided in Text S1 in the supplemental material (section 4), the numerical solution of the above-described system of equations for different values of *S*/*V* predicts the time evolution of the intracellular antibiotic concentration (Fig. 3B), where the steady-state concentration of the antibiotic decreases with decreasing *S*/*V* (Fig. 3C). Here, for simplicity, it is assumed that the antibiotic binds irreversibly to the substrate, *k*_off_ = 0 h^−1^. Changes in *k*_off_ do not strongly impact the dependence of the antibiotic concentration on *S*/*V* (Fig. S1B). Since all antibiotics, apart from membrane-targeting ones, decrease bacterial *S*/*V*, these results point toward an adaptive strategy to reduce intracellular antibiotic concentrations via shape changes.

10.1128/mbio.00659-22.1 TEXT S1 Details of the mathematical model and parameter values for model simulations, organized into four sections: (1) mathematical model for intracellular antibiotic dynamics, (2) mathematical model for the dynamics of membrane-targeting antibiotics, (3) single-cell growth and shape dynamics under treatment with ribosome-targeting antibiotics, and (4) simulation protocol and choice of model parameters. Download Text S1, PDF file, 0.1 MB. Copyright © 2022 Ojkic et al. 2022 Ojkic et al. https://creativecommons.org/licenses/by/4.0/ This content is distributed under the terms of the Creative Commons Attribution 4.0 International license.

To conceptually understand these numerical results, we note that the flux balance is reached at steady state such that

(3) $$\overset{\text{influx}}{\overset{︷}{(a_{\text{out}}-a_{\text{in}})P_{\text{in}}\frac{S}{V}}}=\underset{\text{dilution by growth}}{\underset{︸}{a_{\text{in}}k}}+\underset{\text{depletion by target binding}}{\underset{︸}{k_{x}}}+\underset{\text{efflux}}{\underset{︸}{a_{\text{in}}P_{\text{out}}\frac{S}{V}}}\;$$

in the limit of strong antibiotic-substrate binding (Text S1, section 1). Therefore, the strategy to decrease *S*/*V* upon antibiotic treatment (Fig. 2) results in a reduction in the antibiotic influx term on the left-hand side of equation 3. In the case where efflux pumps are effective, where *P*_out_ is >0, *S*/*V* reduction also leads to an overall decrease in antibiotic efflux.

To quantify the effect of *S*/*V* reduction on the intracellular antibiotic dilution (Fig. 3C), we introduce the antibiotic dilution factor $\text{δ}\equiv |\Delta a_{\text{in}}|/a_{\text{out}}$, defined as the absolute change in the intracellular antibiotic concentration (Δ*a*_in_) relative to the extracellular concentration, as *S*/*V* is varied between a chosen minimum, (*S*/*V*)_min_, and a maximum value, (*S*/*V*)_max_. The dilution factor is thus dependent on the variation in *S*/*V*, growth rate *k*, influx permeability *P*_in_, and outflux permeability *P*_out_ (Fig. 3D; Fig. S2). The maximum dilution is obtained for *P*_out_ = 0 (Fig. 3C and D), while a lesser dilution is obtained for a higher *P*_out_ (Fig. S2D). To determine the maximum δ due to shape variations, we optimize *P*_in_ and *k* for *P*_out_ = 0. We find that δ is maximized when $P_{\text{in}}\sqrt{{\left(S/V\right)}_{\text{min}}{\left(S/V\right)}_{\text{max}}}=k$ (Fig. 3D, dashed line; Text S1, section 1), given by

(4) $${\text{δ}}_{\text{max}}=\frac{\sqrt{{\left(\frac{S}{V}\right)}_{\text{max}}}-\sqrt{{\left(\frac{S}{V}\right)}_{\text{min}}}}{\sqrt{{\left(\frac{S}{V}\right)}_{\text{max}}}+\sqrt{{\left(\frac{S}{V}\right)}_{\text{min}}}}$$

Thus, the maximum value of the dilution factor is dependent only on the surface-to-volume ratios before and after antibiotic application. The above-described equation predicts a maximum of an ~15% dilution in the intracellular antibiotic concentration for cephalexin-treated E. coli, where (*S*/*V*)_max_ and (*S*/*V*)_min_ are taken to be the surface-to-volume ratios for the untreated cell and the antibiotic-treated cell, respectively. Similarly, meropenem-treated A. baumannii cells undergo a maximum of a 22% dilution in the intracellular antibiotic concentration. Antibiotic dilution mediated by changes in *S*/*V* could provide a significant fitness advantage for antibiotics with steep growth inhibition curves (29, 30) since a small reduction in the antibiotic concentration via shape variation could lead to a significant increase in the bacterial growth rate. For spherocylindrical cells of widths *w*_min_ and *w*_max_ before and after antibiotic treatment, the maximum dilution factor can be approximated as

(5) $${\text{δ}}_{\text{max}}\approx \frac{\sqrt{w_{\text{max}}}-\sqrt{w_{\text{min}}}}{\sqrt{w_{\text{max}}}+\sqrt{w_{\text{min}}}}$$

suggesting that the maximum antibiotic dilution depends predominantly on cell width for rod-shaped bacteria (Text S1, section 1).

10.1128/mbio.00659-22.3 FIG S2 Antibiotic dynamics for different parameter values. (A) Antibiotic dynamics for different *S*/*V* values. Data are similar to those in Fig. 3A but for a *k*_on_ of 1 μM^−1^ h^−1^ and a *k*_off_ of 1 h^−1^. (B) Similar to panel A but with a *k*_on_ of 1 μM^−1^ h^−1^ and a *k*_off_ of 10 h^−1^. For the full set of parameters, see Text S1, section 4, in the supplemental material. For this set of parameters, the antibiotic off rate has a marginal effect on antibiotic dynamics inside the cell. (C) Heat map of antibiotic dilutions (δ) for different values of *k* and *P*_in_, setting *P*_out_ as 0.1 μm h^−1^, (*S*/*V*)_min_ as 3 μm^−1^, and (*S*/*V*)_max_ as 15 μm^−1^. (D) Maximum possible antibiotic dilution (δ_max)_ versus *P*_out_. Here, the growth rate is kept fixed (*k *= 1 h^−1^). The maximum possible antibiotic dilution decreases with increasing *P*_out_ and has the highest value for *P*_out_ of 0 μm h^−1^ (Fig. 3D). Download FIG S2, EPS file, 2.1 MB. Copyright © 2022 Ojkic et al. 2022 Ojkic et al. https://creativecommons.org/licenses/by/4.0/ This content is distributed under the terms of the Creative Commons Attribution 4.0 International license.

In contrast to cytosolic antibiotics, when E. coli cells are treated with membrane-targeting and membrane transport-targeting antibiotics, the cell surface area and volume decreased (Fig. S1), whereas the surface-to-volume ratio increased (Fig. 2A). The decrease in surface area is expected since these antibiotics induce membrane damage or a reduction in membrane synthesis. However, the benefits of an increase in *S*/*V* could be interpreted as follows. If the membrane-targeting antibiotic binds directly to the membrane, the dynamics of the surface-bound antibiotic is given by

(6) $$\frac{\text{d}a_{\text{on}}}{\text{d}t}=\underset{\text{binding to membrane}}{\underset{︸}{P_{\text{on}}a_{\text{out}}\frac{V}{S}}}-\underset{\text{unbinding from membrane}}{\underset{︸}{P_{\text{off}}a_{\text{on}}}}-\underset{\text{dilution by growth}}{\underset{︸}{ka_{\text{on}}}}$$

where *a*_on_ is the surface density of the antibiotic and *P*_on_ and *P*_off_ are the rates of binding to and unbinding from the membrane surface, respectively (see Text S1, section 2, for derivation). The first term on the right-hand side of equation 6 represents the total amount of antibiotic molecules (*a*_out_ *V*) that can bind to the membrane per unit surface area and per unit time with a probability *P*_on_. Therefore, an increase in *S*/*V* decreases the density of membrane-bound antibiotics, thereby providing a beneficial morphological adaptation for countering the action of the antibiotic.

## MECHANISMS OF CELL SHAPE CHANGES BY ANTIBIOTIC ACTION

The mechanisms by which antibiotics induce cell shape changes are specific to the type of antibiotic treatment (13). DNA-targeting antibiotics induce an SOS response that inhibits cell division, resulting in longer filamentous cells (13, 29) (Fig. 2). Cell wall-targeting antibiotics such as β-lactams bind to penicillin binding proteins and inhibit peptidoglycan synthesis. This affects septal cell wall synthesis, leading to longer filamentous cells, while the inhibition of peptide cross-linking by β-lactams can result in wider cells with a lower surface-to-volume ratio. Ribosome-targeting antibiotics inhibit translation, inducing a variety of shape changes depending on the nutrient availability in the growth environment (15, 18, 20). Below, we specifically focus on the case of ribosome-targeting antibiotics, for which the biochemical reactions are well characterized (31), in order to elucidate how the coupling between cell growth, shape, and protein synthesis regulates the cellular morphological response.

**FIG 4:**
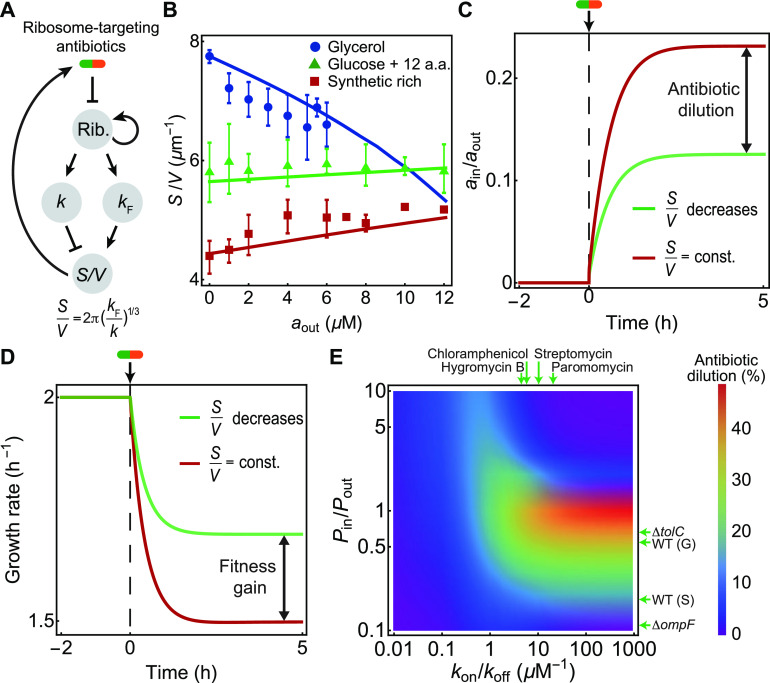
Interplay between cell shape and translation-inhibiting antibiotics. (A) Schematic diagram of the feedback pathways connecting ribosomal translation to cell shape and antibiotic transport. Ribosomes promote growth (*k*), which in turn decreases the surface-to-volume ratio (*S*/*V*), and an increase in the division protein production rate (*k_F_*) increases the surface-to-volume ratio. *S*/*V* promotes antibiotic influx (Fig. 1B; equation 1). (B) *S*/*V* versus the chloramphenicol concentration in E. coli (20, 33). Experimental data under different nutrient conditions are shown as scatter points. Error bars represent the standard errors of the means, with a maximum of four replicates under each growth condition. Simulation predictions are shown as solid lines. a.a., amino acids. (C) Relative antibiotic concentration inside the cell versus time obtained by model simulations for two cases, (i) *S*/*V* = constant = 5 μm^−1^ (red) and (ii) *S*/*V* decreases from 5 to 3 μm^−1^ (green), via the pathway shown in panel A. Here, *a*_out_ is 5 μM, and *P*_in_/*P*_out_ is 1. (D) Bacterial growth rate versus time obtained from simulations for two different cases as described above for panel C. The decrease in *S*/*V* results in a fitness gain. (E) Heat map of antibiotic dilution factors predicted from simulations as functions of membrane permeability ratios (*P*_in_/*P*_out_) and the ratio of antibiotic-ribosome binding and unbinding rates (*k*_on_/*k*_off_). The antibiotic dilution was calculated when the bacterial *S*/*V* was altered from 15 to 3 μm^−1^, as in Fig. 3C. Membrane permeability ratios shown with horizontal arrows were estimated (see Text S1, section 1, in the supplemental material) for growing wild-type (WT) (G) and stationary-phase wild-type (S) E. coli cells and OmpF porin-deficient (Δ*ompF*) and efflux-pump deficient (Δ*tolC*) cells (35). Experimentally measured *k*_on_/*k*_off_ values for different ribosome-targeting antibiotics are shown with vertical arrows (58–60).

One of the most commonly used ribosome-targeting antibiotics is chloramphenicol (CHL), which inhibits bacterial translation. When actively translating ribosomes are blocked by CHL, cells synthesize new ribosomes in excess to counterbalance their inactivation (32). This increase in ribosome abundance requires bacteria to strategically focus their ribosomal resources toward growth rather than division, causing an increase in the cell volume (33). Interestingly, when E. coli cells are exposed to nutrient or CHL perturbations, the bacterial cell aspect ratio remains constant at ~4 (17), implying a simple scaling relation between surface area and volume, *S *= 2π*V*^2/3^, yielding *S*/*V* = 2π*V*^−1/3^ (17). Therefore, if the bacteria increase their volume upon CHL treatment, due to excess ribosome synthesis, *S*/*V* would then decrease (Fig. 4A and B).

Experimental data, however, show that *S*/*V* increases or decreases upon chloramphenicol treatment depending on nutrient availability in the growth environment (20) (Fig. 4B). In nutrient-rich medium, *S*/*V* decreases upon chloramphenicol treatment, whereas in nutrient-poor medium, *S*/*V* increases upon chloramphenicol treatment (Fig. 4B). To interpret these results and quantitatively explain the data, we applied a previously developed whole-cell model for bacterial growth and division control (33) in the context of translation inhibition. In this model, the cell volume grows exponentially at a rate, *k*, whereas division proteins are synthesized at a volume-specific rate, *k_F_*. The cell divides when a threshold amount of division proteins is synthesized, resulting in the relation *V *=* k*/*k_F_*. This implies for E. coli cells that *S*/*V* = 2π(*k_F_*/*k*)^1/3^ (Fig. 4A; see also Text S1, section 3, in the supplemental material). The data in Fig. 4B can now be interpreted using the framework of cellular resource allocation (34). Upon treatment with chloramphenicol, cells upregulate ribosome synthesis to compensate for ribosome inhibition by chloramphenicol (32). The excess ribosomes that are produced are allocated differentially to growth (*k*) or division protein synthesis (*k_F_*) depending on nutrient availability. In nutrient-rich medium, cells allocate more ribosomes to division than growth (*k*/*k_F_* decreases), resulting in smaller cells and higher *S*/*V*. The increase in *S*/*V* provides an adaptive benefit by importing more nutrients to counter growth inhibition by the antibiotic. In contrast, in nutrient-poor medium, cells tend to allocate more ribosomes to growth than division (*k*/*k_F_* increases), resulting in larger cells and smaller *S*/*V*. A reduction in *S*/*V* reduces antibiotic influx, which is more beneficial for survival if nutrient availability is low.

To estimate the amount of antibiotic dilution due to cell shape changes, we simulated the whole-cell model to evolve the coupled dynamics of cell size and shape, division proteins, ribosomes, and the antibiotic (Text S1, section 3) (33). Using parameters benchmarked for E. coli cells, our model can quantitatively capture the experimentally observed trends for the dependence of *S*/*V* on the antibiotic concentration in different nutrient environments (Fig. 4B, solid lines). We find that under conditions where *S*/*V* remains unchanged, the intracellular antibiotic concentration is higher and the growth rate is lower than in the case where *S*/*V* spontaneously decreases over time (Fig. 4C and D). Our simulations revealed that the maximum antibiotic dilution was obtained for antibiotics with high affinity constants (*k_A_* = *k*_on_/*k*_off_ > 1 μM^−1^) typical for aminoglycosides: hygromycin B (*k_A_* = 5 μM^−1^), chloramphenicol (*k_A_* = 5.8 μM^−1^), streptomycin (*k_A_* = 10 μM^−1^), and paromomycin (*k_A_* = 19 μM^−1^). Depending on the ratio of the membrane permeability constants (*P*_in_/*P*_out_), the antibiotic dilution factor is nonmonotonic and reaches a maximum for a *P*_in_/*P*_out_ value of ~1 (Fig. 3C and Fig. 4E). Since aminoglycosides are predominantly transported inside the cell by porins, we estimated the permeability coefficients for the fluorescent antibiotic ofloxacin, which is also translocated by porins (35). By analyzing time traces of fluorescent-antibiotic accumulation inside the cell, we estimated that growing E. coli cells readily accumulate antibiotics, with a *P*_in_/*P*_out_ value of ~0.54, while for stationary-phase cells, the *P*_in_/*P*_out_ value is ~0.11. Interestingly, for bacterial cells lacking porins (Δ*ompF*), *P*_in_/*P*_out_ is ~0.18, and for bacteria lacking efflux pumps (Δ*tolC*), *P*_in_/*P*_out_ is ~0.66 (Text S1, section 1). Therefore, these results suggest that the antibiotic dilution by changes in *S*/*V* could approach the maximum value achieved for *P*_in_ ≈ *P*_out_ and a large *k*_on_/*k*_off_ (Fig. 4E).

## CONCLUSION

In this article, we used quantitative modeling and morphological data analysis to demonstrate that bacteria can develop antibiotic resistance purely by cell shape changes. By analyzing cell size and shape data for three different bacterial species treated with 46 different antibiotics, we find that bacterial cells robustly reduce surface-to-volume ratios when treated with cytosolic antibiotics. A reduction in *S*/*V* comes with the adaptive benefit of decreasing antibiotic influx, which can effectively dilute the concentration of intracellular antibiotics. For membrane-bound antibiotics that induce membrane damage, an increase in *S*/*V* promotes drug dilution, in agreement with experimental observations. By developing a whole-cell model for bacterial growth, we further show how antibiotic-induced shape changes can provide fitness advantages in the presence of antibiotics and compare the model predictions with experimental data.

Since antibiotic dilution predominantly depends on cell width (equation 5), a central question is how width is determined (36–38) and how fast width is remodeled in response to antibiotic treatments. During steady-state growth, cell width is one of the most tightly controlled cellular parameters in both E. coli and B. subtilis (39, 40) such that fluctuations in width are restored within 4 to 5 generations (17), commensurate with the typical time necessary for bacterial width remodeling (41). Under non-steady-state conditions, the cell width reaches a new equilibrium only within a few generations after nutrient shifts (42) or antibiotic exposure (15, 18). Cell width control is achieved by cell wall synthesis machinery such as MreB, RodZ, and penicillin binding proteins (43) as well as by physical forces, including mechanical stress on the cell envelope and osmotic pressure (44–46). Understanding the mechanisms of cell shape regulation therefore requires an integrative approach, combining cellular mechanics and biochemical regulation.

In addition to the surface-to-volume ratio, recent work found that *Vibrio* bacteria can also alter curvature when exposed to antibiotics (18). In particular, when C. crescentus is treated with sub-MICs of chloramphenicol (CHL), the cell becomes more curved, and the cell width increases (18). Immediately upon CHL treatment, the cell growth rate decreases, but over ~10 generations, the growth rate is gradually restored to the preantibiotic level. While the cell *S*/*V* decreases during CHL exposure, the contribution of a lowered *S*/*V* cannot solely explain the almost full growth rate recovery. This adaptive response via cell shape changes can be explained by a model of negative feedback between the cellular growth rate and cell envelope mechanical tension (18). Translation inhibition by CHL reduces the rate of synthesis of cell envelope material, which leads to an initial fast drop in the growth rate. The reduced rate of surface area synthesis also reduces the effective tension that works against the compressive bending forces acting on the cell surface. As a result, reduced tension leads to cell surface bending until a new mechanical equilibrium is reached with a higher curvature. Lower cell envelope tension promotes cell wall synthesis (45), thereby increasing the growth rate to its prestimulus value. Future experiments are needed to further test the role of cell curvature and wall tension in the maintenance of growth rate homeostasis. In particular, it will be interesting to test whether *Caulobacter* mutant cells with straight morphologies are poor at adapting to chloramphenicol (47) or whether *Vibrio* bacteria adapt better than straight rod-shaped cells.

A reduction in the surface-to-volume ratio not only decreases antibiotic influx but also leads to reduced nutrient influx that may in turn decrease cellular metabolic activity. In recent work, Lopatkin et al. showed that metabolism plays a crucial role in the bacterial response to antibiotics such that cells with decreased metabolic activities are more antibiotic resistant (48). Metabolic mutations in response to antibiotic exposure suggest adaptive mechanisms in central carbon and energy metabolism. Interestingly, some of the advantageous metabolic mutations that mitigate antibiotic susceptibility have been identified in >3,500 clinically relevant pathogenic E. coli isolates (48). These findings point toward a new pathway of antibiotic resistance mediated by mutations in the core metabolic genes. Decrease in cell surface-to-volume ratio may act in synergy with the metabolic slowdown to confer stronger antibiotic resistance.

In synergy with shape changes, bacteria can actively regulate the antibiotic concentration inside the cell by controlling porin and efflux pump expression (Fig. 1B) (49, 50). Cell wall-targeting antibiotics, such as β-lactams, that disrupt the stability of the peptidoglycan meshwork are translocated by OmpF porins to induce the envelope stress response (Cpx) (51). The activation of the Cpx system decreases *ompF* expression (52), creating a negative-feedback loop (Fig. 1B), resulting in lower porin numbers and lower inward membrane permeability (*P*_in_). Similarly, when E. coli cells are exposed to DNA-targeting antibiotics that are also translocated inside the cell by OmpF, the expression level of *ompF* decreases within 30 to 120 min after antibiotic treatment (49). A reduction in porin numbers will act in synergy with a reduction of *S*/*V* to confer stronger resistance phenotypes. In addition to controlling antibiotic influx, bacteria can decrease the intercellular antibiotic concentration through the overexpression of efflux pumps (49, 53, 54) (Fig. 1B). In the future, time-lapse experiments are necessary to reveal the time scales associated with the onset and completion of morphological transformation under antibiotic perturbations and how these time scales compare with changes in the expression profiles of proteins responsible for regulating antibiotic influx and efflux. These studies would be essential to quantify the contributions of the different resistance pathways and their synergistic effects responsible for increasing bacterial fitness.
